# 10-undecynoic acid is a new anti-adherent agent killing biofilm of oral *Streptococcus* spp.

**DOI:** 10.1371/journal.pone.0214763

**Published:** 2019-04-18

**Authors:** Anna Goc, Waldemar Sumera, Aleksandra Niedzwiecki, Matthias Rath

**Affiliations:** Department of Infectious Diseases, Dr. Rath Research Institute BV, Santa Clara, California, United States of America; Laurentian, CANADA

## Abstract

In the search for novel agents against oral pathogens in their planktonic and biofilm form, we have focused our attention on 10-undecynoic acid as the representative of the acetylenic fatty acids. Using macro-broth susceptibility testing method we first established MIC value. Next, the MBC value was determined from a broth dilution minimum inhibitory concentration test by sub-culturing it to BHI agar plates that did not contain the test agent. Anti-biofilm efficacy was tested in 96-well plates coated with saliva using BHI broth supplemented with 1% sucrose as a standard approach. Based on obtained results, MIC value for 10-undecynoic acid was established to be 2.5 mg/ml and the MBC value to be 5 mg/ml. The MBIC_90_ showed to be 2.5 mg/ml, however completed inhibition of biofilm formation was achieved at 5.0 mg/ml. MBBC concentration revealed to be the same as MBC value, causing approximately 30% reduction at the same time in biomass of pre-existing biofilm, whereas application of 7.0 mg/ml of 10-undecynoic acid crossed the 50% eradication mark. Strong anti-adherent effect was observed upon 10-undecynoic acid application at sub-MBC concentrations as well, complemented with suppression of acidogenicity and aciduricity. Thus, we concluded that 10-undecynoic acid might play an important role in the development of alternative or adjunctive antibacterial and anti-biofilm preventive and/or therapeutic approaches.

## Introduction

Dental caries is one of the most prevalent diseases in humans provoked by the disbiose in oral biofilm due to frequent sugar intake [1–4]. Despite scientific and clinical advances as well as public awareness focused on the importance of oral hygiene and dental health, dental caries still remains the main cause of tooth loss in children and is a common problem affecting young and older adults [1]. Thus, it is a global public health problem to be managed in modern times. Although in recent years the occurrence of dental caries in developed countries has declined, the opposite trend is taking place in developing countries [5,6].

The species often collectively referred to as the mutans streptococci (MS) have been primarily associated with the development of dental caries in humans [7–9]. Among them, *Streptococcus mutans* and *Streptococcus sobrinus* were found to be the most frequently isolated from human dental plaques and recognized as the principal bacteria of dental caries. Their natural habitat is the human mouth and the long list of scientific and clinical reports have conclusively demonstrated that these major cariogenic organisms contribute to the formation of the dental plaque (commonly called oral biofilm) and have the ability to successfully compete with commensal species by the means of higher tolerance to low pH [8,10,11]. It is important to mention, however, that caries may occur even when *Streptococcus mutans* and *Streptococcus sobrinus* are not identified in cariogenic biofilm. Their ability to adhere, aggregate, and form and sustain a polysaccharide-enclosed biofilm is a key to their survival and persistence in the oral cavity [12,13]. Primary attachment to solid tooth surfaces is reversible and mediated by MS fimbriae recognizing and binding to enamel pellicle. This sucrose-independent mechanism is observed between the adhesive particles of MS and saliva agglutinins, and is involved in the process of adhesion promoting cohesion. Irreversible attachment follows after as a result of the synthesis of glucans and fructans, from sucrose as their only source, by glucosyltransferase (GTF) and fructosyltransferase (FTF), in combination with glucan-binding proteins (GBPs) mediating the binding of bacteria to these extracellular polysaccharides (EPS) [14–16]. Hence, suppression of biofilm of oral MS may be an appealing preventive approach to oral cavities. However, the cariogenic process is the consequence of several subsequent events. To develop dental caries it is necessary to accumulate biofilm and expose it frequently to carbohydrates, fermented by oral pathogenic bacteria. If a person does not consume fermentable carbohydrates, even having biofilm, he/she will never develop the dental caries [2,3]. Thus, targeting other most often documented characteristics of the virulence of MS, which is a metabolism of carbohydrates that leads to acidification of dental plaque (acidogenicity), and their capacity to tolerate an acidic environment (aciduricity) is a key in prevention. These major factors are directly related to the pathogenesis of dental caries by the virtue of demineralization of the tooth enamel [16,17]. Acidogenicity is one of the factors that allows the ecological changes characterized by outgrowth of species that are similarly acidogenic and aciduric, such as *S*. *mutans and S*. *sobrinus*, resulting in elevated quantities of these organisms in dental plaques. It seems that lactic acid is the most important acidic metabolite involved in etiology of dental caries since it is the strongest acid abundantly produced by lactate dehydrogenase (LDH) of MS [11,18]. Aciduricity is another fundamental factor of MS in which membrane-bound F-ATPase works as a pump to transport protons from cells and maintains tolerable pH when the external pH falls to 4.0 or below. Due to the acid production and acid resistance, *S*. *mutans* and *S*. *sobrinus* gain an advantage over other non-cariogenic bacteria and are less susceptible to lethal environmental conditions associated with enhanced glycolytic capacities and increased activity of the proton-translocating F-ATPase during caries development [11,19,20]. Nevertheless, it is worth mentioning that MS have a complex acid tolerance response (ATR) involving many genes and proteins, where F-ATPase is one of them [21]. Since mutants of *S*. *mutans*, deficient in specific virulence factors, are more sensitive to environmental stress and less cariogenic than their parent strains [22,23], this would suggest that suppression of virulence-associated genes and enzymes could be appealing for the prevention of dental caries.

Mechanical plaque removal as well as the broadly and commonly use of different kinds of antimicrobial agents, and antibiotics when necessary, are well known factors controlling dental plaque development and are effective strategies to prevent dental caries progression [8,24–28]. Despite the reported therapeutic potential of many of these chemical and natural compounds, little is known about the efficacy of fatty acids as antimicrobial and anti-caries compounds. We have screened several of them and noticed that one of them, i.e., acetylenic fatty acid 10-UDYA, known highly selective irreversible inhibitor of hepatic *ω*- and *ω*-1-hydroxylases [29], revealed outstanding bacteriostatic and bactericidal efficacy *in vitro*. In our investigation we explored further this feature and for the first time demonstrate here insightful evaluation of this compound as an anti-biofilm agent. This compound upsets acid tolerance of *S*. *mutans* to low pH *in vitro* and has excellent anti-adherent properties. Also, its fast biofilm penetrating property could be an additional contributor to the very effective killing effect of pre-existing biofilm. Our work represents systematic investigation of the effects of 10-UDYA on virulence factors of MS, although, the obtained results warrant more studies on practical applicability of this compound in oral health.

## Materials and methods

### Bacterial strains and media

The majority of the experiments were performed with *Streptococcus mutans* UA159 serotype c (ATCC 700610), and *Streptococcus sobrinus* SL1 (ATCC 33478). Selection and focus on these two strains was based on scientific and clinical reports identifying these MS as well-established, virulent, and biofilm forming bacteria [30,31]. These two strains were implicated as primary bacteria of dental caries based on elevated quantities of these organisms found in dental plaque from caries-active subjects. *Streptococcus mutans* NCTC 10449 (ATCC 25175) and *Streptococcus sobrinus* NIDR 6715–15 (ATCC 27352) were used in the experiments aimed to establish basic microbiological parameters such as MIC (minimal inhibitory concentration), MBC (minimal bactericidal concentration), MBIC (minimal biofilm inhibitory concentration), MBBC (minimal biofilm biocidal concentration), and MBEC (minimal biofilm eradication concentration) values, and to support results obtained from experiments performed with *Streptococcus mutans* UA159 and *Streptococcus sobrinus* SL1. The stocks of all strains were cultured as recommended by manufacturer conditions, i.e., BHI (Brain Heart Infusion) broth (Remel, San Diego, CA) without antibiotics at 37°C with 5% CO_2_ in sterile two-position cap 5 ml polypropylene test tubes. All strains were stored in -80°C in BHI broth containing 30% glycerol.

### Preparation of 10-undecynoic acid for susceptibility testing

A stock solution (100 mg/ml) of 10-undecynoic (10-UDYA) (Sigma, St. Louis, MO) was prepared in ≥ 99.9% DMSO (Sigma, St. Louis, MO, CA) and sterilized by 0.22 μm syringe filtration and prepared fresh just before each experiment. Due to possible bactericidal effect of a high percentage of DMSO, its added amount to the growth medium was kept ≤ 0.14% (v/v). The appropriate amount of stock solution was then added to either sterile two-position-cap test tubes, or 8-well chambers containing 1 ml of BHI broth, or to 96-well plates containing 0.2 ml of BHI broth to yield final concentrations of 0.25, 0.5, 1.0, 2.5, 5.0, and 7.0 mg/ml (1.37, 2.74, 5.49, 13.72, 27.4, and 38.4 mM).

### Evaluation of the bacteriostatic and bactericidal effects of 10-undecynoic acid against the planktonic form of oral *Streptococcus* spp

Growth inhibition of *Streptococcus* spp. was tested using a standard macro-dilution method to establish MIC value [32]. Briefly, sterile 3 ml two-position-capped test tubes containing 1 ml BHI broth with 1 x 10^6^ CFU/ml of the homogenous bacterial suspension were supplemented with the 10-UDYA. The tubes were then incubated at 37°C with 5% CO_2_ and growth inhibition as a decrease in the optical density (OD_600_) was monitored at regular intervals for up to 24 h. The entire experiment was repeated three times for each strain. Control cultures were treated with DMSO (≤ 0.14% v/v). The MBC values were determined from a broth dilution minimum inhibitory concentration test by sub-culturing the bacterial cell samples removed at 0, 1, 3, 6, 12, and 24 h, and plated onto BHI agar plates that did not contain 10-UDYA. The plates were then incubated at 37°C with 5% CO_2_ and bacterial re-growth was assessed after 24 h. A bacteriostatic effect was defined as 2-log_10_ CFU/ml, whereas a bactericidal effect was defined as a 3-log_10_ CFU/ml decrease from the original inoculum [33]. All experiments were conducted three times independently and each one in three replicates. As a positive control amoxicillin (Sigma, St. Louis, MO) at the concentration range between 2–500 μg/ml was chosen, which still is the drug of choice in treating dental infections [34].

### Evaluation of the effect of 10-undecynoic acid on biofilm of oral *Streptococcus* spp

Biofilm preventive effect of the 10-UDYA focused on establishing MBIC values against *Streptococcus* spp. was evaluated by the alamarBlue and commonly used crystal violet (CV) staining methods. Briefly, 1 x 10^7^ CFU/ml from homogeneous bacterial culture in BHI broth containing 1% sucrose, as a standard approach [32], was inoculated into 96-well plates coated with human saliva and supplemented with the 10-UDYA. In addition, a supportive experiment was performed in which instead of human saliva coated plates, human saliva coated artificial teeth were used (Azdent, Baku, Azerbaijan). Control wells were treated with DMSO (≤ 0.1% v/v). All plates were then incubated at 37°C with 5% CO_2_ for 24 h. Next, all wells were either stained with ready-to-use alamarBlue dye (where resazurin, a non-fluorescent indicator dye, is converted to bright red-fluorescent resorufin via the reduction reactions of metabolically active cells and the amount of fluorescence produced is proportional to the number of living cells) for evaluating cellular metabolic activity according to published results [35] and the manufacturer’s recommendation (Thermo Fisher, Waltham, MA), or fixed with 0.2 ml of cold methanol-formalin (1:1) for 30 min. and stained with 0.1 ml of crystal violet (0.1%) for 10 min. The biofilms stained with alamarBlue were incubated at 37°C with 5% CO_2_ for 30–60 min. and fluorescence intensity was measured using excitation wavelength 535 nm and emission wavelength 595 nm. The biofilms stained with CV were thoroughly washed three times with 1 x PBS (phosphate-buffered saline), and 0.2 ml of methanol was added to each well to extract a dye, then the absorbance was measured at 595 nm using a spectrophotometer (Molecular Device, Spectra Max 340). In addition, 1 x 10^7^ CFU/ml from homogeneous bacterial culture in BHI broth containing 1% sucrose was inoculated into human saliva coated 8-well chambers and supplemented with the 10-UDYA. Control wells were treated with DMSO (≤ 0.14% v/v), and incubated at 37°C with 5% CO_2_ for 24 h. Next, all wells were fixed with 0.5 ml of cold formalin acetic acid mixture for 20 min., followed by staining with 0.5 ml of LIVE/DEAD^®^ BacLight™ Bacterial Viability staining mixture for 15 min. in the dark, according to the manufacturer’s recommendation (Thermo Fisher, Waltham, MA). Pictures were immediately taken from untreated and treated mounted slides with a fluorescence microscope (Nikon, Eclipse E600) using excitation wavelength 488 nm.

Biocidal and eradicative effect of 10-UDYA against pre-existing biofilms of *Streptococcus* spp. was evaluated (both qualitatively and quantitatively) by the alamarBlue and crystal violet (CV) staining methods, and supported by LIVE/DEAD^®^ BacLight™ Bacterial Viability staining with fluorescent microscopy (Nikon, Eclipse E600). To establish MBBC and MBEC values, 1 x 10^7^ CFU/ml from homogeneous bacterial culture in BHI broth containing 1% sucrose, as a standard approach, was inoculated into human saliva coated 96-well plates or 8-well chambers. Additionally, a supportive experiment was performed in which instead of human saliva coated plates, human saliva coated artificial teeth were used (Azdent, Baku, Azerbaijan). After 24 h biofilms were washed with 1 x PBS and treated with 10-UDYA. Control wells were treated with DMSO (≤ 0.14% v/v). All plates were then incubated at 37°C with 5% CO_2_ for up to 24 h. Next, all wells were stained with ready-to-use alamarBlue dye for 30–60 min., and 0.1 ml of crystal violet or 0.5 ml of LIVE/DEAD^®^ BacLight™ Bacterial Viability staining mixture for 10–15 min. according to the manufacturer’s recommendation (Thermo Fisher, Waltham, MA). All experiments were conducted three times independently and each one in three replicates.

In addition, a set of experiments was performed in which 1 x 10^7^ CFU/ml from homogeneous bacterial culture in BHI broth containing 1% sucrose, as a standard approach, was inoculated into human saliva coated 96-well plates. After 24 h biofilms were washed with 1 x PBS and treated with 10-UDYA. Control wells received DMSO (≤ 0.14% v/v). All plates were then incubated at 37°C with 5% CO_2_ for up to 24 h. Next, the treatment with 10-UDYA was removed, all wells were washed with 1 x PBS, and BHI broth with 1% of sucrose without test agent was applied for 16 h to allow the recovery of biofilm. After that, the wells were stained with ready-to-use alamarBlue dye to evaluate cellular health, or fixed with 0.2 ml of cold methanol-formalin (1:1) for 30 min. and stained with 0.1 ml of crystal violet (0.1%) for 10 min. The biofilms stained with alamarBlue were incubated at 37°C with 5% CO_2_ for 30–60 min., and fluorescence intensity was measured using excitation wavelength 535nm and emission wavelength 595 nm. The biofilms stained with CV were carefully washed three times with 1 x PBS (phosphate-buffered saline), and 0.2 ml of methanol was added to each well to extract a dye, and the absorbance was measured at 595 nm using a spectrophotometer (Molecular Device, Spectra Max 340). Also, for further confirmation, an aliquot of 0.1 ml of the biofilm suspensions treated with different concentrations of 10-UDYA for 24 h, respectively, were ten-fold serially diluted in saline solution, and 20 μl of each dilution was plated on BHI agar plates, incubated at 37°C for 24 h, and the counts of the colony forming unit (CFU) were performed. All experiments were conducted three times independently and each one in three replicates. As a positive control amoxicillin (Sigma, St. Louis, MO) at the concentration range between 2–500 μg/ml was chosen, which still is the most common antibiotic used by dentists in treating dental infections [34].

### Biofilm analyses

For imaging the exopolysaccharide (EPS) presence on pre-existing biofilms, *Streptococcus* spp. biofilms were stained with 1 μM Alexa Fluor 633 labeled concanavalin A, and immersed in ultra-pure water for up to one hour, according to published study [36]. Concanavalin A belongs to the lectins that bind selectively to α-glucopyranosyl molecules such as glucans, which are major components of the EPS. The fluorescent dye was excited with HeNe laser (633 nm). For polysaccharides and protein estimations, biofilms of *Streptococcus* spp. were grown in 24-well plates coated with human saliva for 24 h. Next, planktonic bacterial cells were washed out and the biofilms were treated with different concentrations of 10-UDYA for an additional 24 h. The biofilms were then collected by sonication/vortexing in 1 x PBS and the total amount of carbohydrates were determined by the anthrone method with glucose as the standard [37]; whereas the total amount of protein was assessed with a BCA Protein Assay Kit according to manufacturer’s protocol with bovine serum albumin as the standard (Thermo Fisher, Waltham, MA). Another aliquot of the sonicated biofilms suspensions were added to pre-weighed micro-centrifuge tubes and air-dried to assess the biomasses of biofilms, which was determined by the difference between the terminal and initial weight of the micro-centrifuge tubes [37].

The extraction and estimation of the amounts of extracellular polysaccharides, both soluble and insoluble, were performed as previously reported [37,38] from an aliquot of 0.3 ml of the sonicated biofilm suspensions. Briefly, the aliquots of biofilm suspensions were centrifuged at 10,000 g for 10 min. at 4°C. The supernatants were collected and the biofilm pellets were re-suspended and washed twice with sterile water and again centrifuged at 10,000 g for 10 min. to separate water-soluble (supernatant) and water-insoluble polysaccharides (pellet). Three parts of cold methanol were added to each collected supernatant to precipitate the polysaccharide. After a series of centrifugation, the precipitates were air-dried and re-suspended in water, and the amount of water-soluble polysaccharides was measured by the anthrone method, with glucose as the standard. The pellets were dissolved in 1 M NaOH (1 mg of pellet/0.3 ml of 1 M NaOH) and the water-insoluble polysaccharides were quantified in a similar manner as supernatants. The absorbance was measured at 625 nm and the concentrations of water-soluble and water-insoluble polysaccharides were calculated using standard curves. All experiments were conducted three times independently and each one in three replicates.

### Adherence assay

Glass surface adherence assay was performed by the method of Hamada, *et al*. [39]. Briefly, *S*. *mutans* UA159 was grown for 24 h at 37°C at an angle of 30 degrees in glass tubes containing 5 ml of BHI with or without 1% sucrose and various concentrations of 10-UDYA. The control tubes contained BHI (with or without sucrose) and equivalent amounts of DMSO. After incubation, planktonic cells were decanted, and the attached bacterial cells were removed by 0.5 M NaOH. Adherence was quantified by recording changes in OD_600_ as fallows: Percentage adherence = [OD_600_ of adhered cells/OD_600_ of adhered cells+OD_600_ of supernatant cells] x 100. All experiments were conducted three times independently and each one in three replicates.

### Glycolytic pH drop and acid tolerance assay

The effect of 10-UDYA on *S*. *mutans* UA159 glycolysis was measured according to Xu, *et al*. [32] per the method modified from Song, *et al*. [40]. Briefly, *S*. *mutans* UA159 was harvested at mid-logarithmic phase, washed with 0.5 mM potassium phosphate buffer containing 37.5 mM KCl and 1.25 mM MgCl_2_ (pH = 6.5), and re-suspended to OD_600_ = 0.5 in the same solution supplemented with different concentrations of 10-UDYA (0.25–7.0 mg/ml). The control tubes contained equivalent amounts of DMSO. Glucose was added to the mixture to give a final concentration of 1%. The decrease in pH, as a result of glycolytic activity of *S*. *mutans* UA159, was monitored every 30 min. up to 2 h (Mettler-Toledo, Columbus, OH). The effect of 10-UDYA on the acid tolerance of *S*. *mutans* UA159 was determined by measurement of the viability of bacteria after 2 h of exposure at pH = 5.0 [40,41]. *S*. *mutans* UA159 was grown in TYEG (tryptone-yeast extract supplemented with 20 mM glucose) medium until the bacterial cells reached the mid-logarithmic phase equal to OD_600_ = 0.5. The cells were collected by centrifugation and re-suspended to OD_600_ = 0.2 in TYEG medium buffered with 20 mM phosphate-citrate buffer (pH = 5.0) containing different concentrations of 10-UDYA, and incubated at 37°C for 2 h. The control mixture contained no 10-UDYA. Samples were removed before and after incubation at pH = 5.0 for viable counts on BHI agar plates. All experiments were conducted three times independently and each one in three replicates.

### Biochemical assays

For F-ATPase assays of *S*. *mutans* UA159, pre-existing biofilms were treated with different concentrations of 10-UDYA and permeabilized according to the method described by Belli, *et al*. [42]. The F_1_F_0_-ATPase activity was determined by the amount of inorganic phosphate released in the reaction mixture of 75 μl of permeabilized cells and 3 ml of 50 mM Tris-maleate buffer (pH 6.0) with 10 mM MgSO_4_. The mixture was then heated to 37°C and the reaction was initiated by adding 30 μl of 0.5 M ATP (pH = 6.0). After 30 min., the reaction was stopped and the released phosphate was determined according to Muñoz, *et al*. [43]. The results were expressed as enzymatic activity relative to the untreated control. All experiments were conducted three times independently and each one in three replicates.

For lactic acid measurement, *S*. *mutans* UA159 pre-existing biofilms were treated with different concentrations of 10-UDYA for 24 h at 37°C. After removing planktonic cells by centrifugation (10,000 g, 5 min., 4°C), the supernatants were decanted to measure lactate concentrations according to the manufacture’s protocol of the Lactate Assay Kit (Sigma, St. Louis, MO). The absorbance at 570 nm was recorded using a microplate spectrophotometer and lactate concentrations were calculated using standard curves. All experiments were conducted three times independently and each one in three replicates.

### Statistical analysis

All data are presented as means ± SD (n = 3). The Student's two-tailed t test was used to determine statistically significant differences set at 0.05 levels. Statistical analysis was performed using GraphPad software.

## Results

### Bacteriostatic and bactericidal effect of 10-undecynoic acid against the planktonic and biofilm form of oral *Streptococcus* spp. *in vitro*

**Fig 1:**
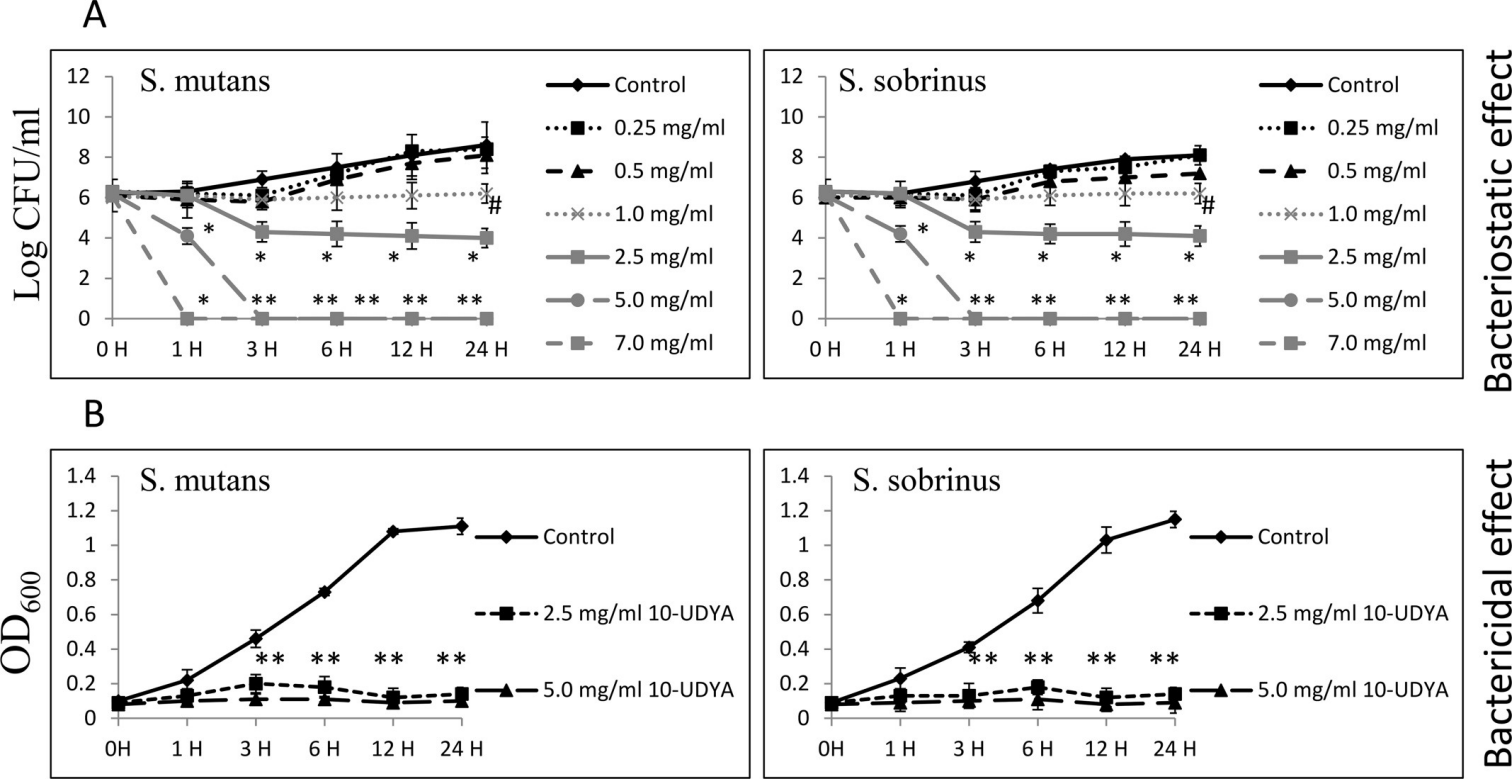
Bacteriostatic and bactericidal efficacy of 10-undecynoic acid on planktonic form of *Streptococcus mutans* UA159 and *Streptococcus sobrinus* SL1. (A) Time-dependent effect of different concentrations of 10-undecynoic acid assessed by plating method up to 24 h. (B) Time-dependent effect of MIC and MBC values of 10-undecynoic acid assessed by macro-dilution method up to 24 h. MBC values were determined from broth macro-dilution minimum inhibitory concentration test by sub-culturing it to BHI agar plates that do not contain the test agent. Control– 0.1% DMSO, 10-UDYA– 10-undecynoic acid, # p<0.05, * p<0.001 compared to control.

**Fig 2:**
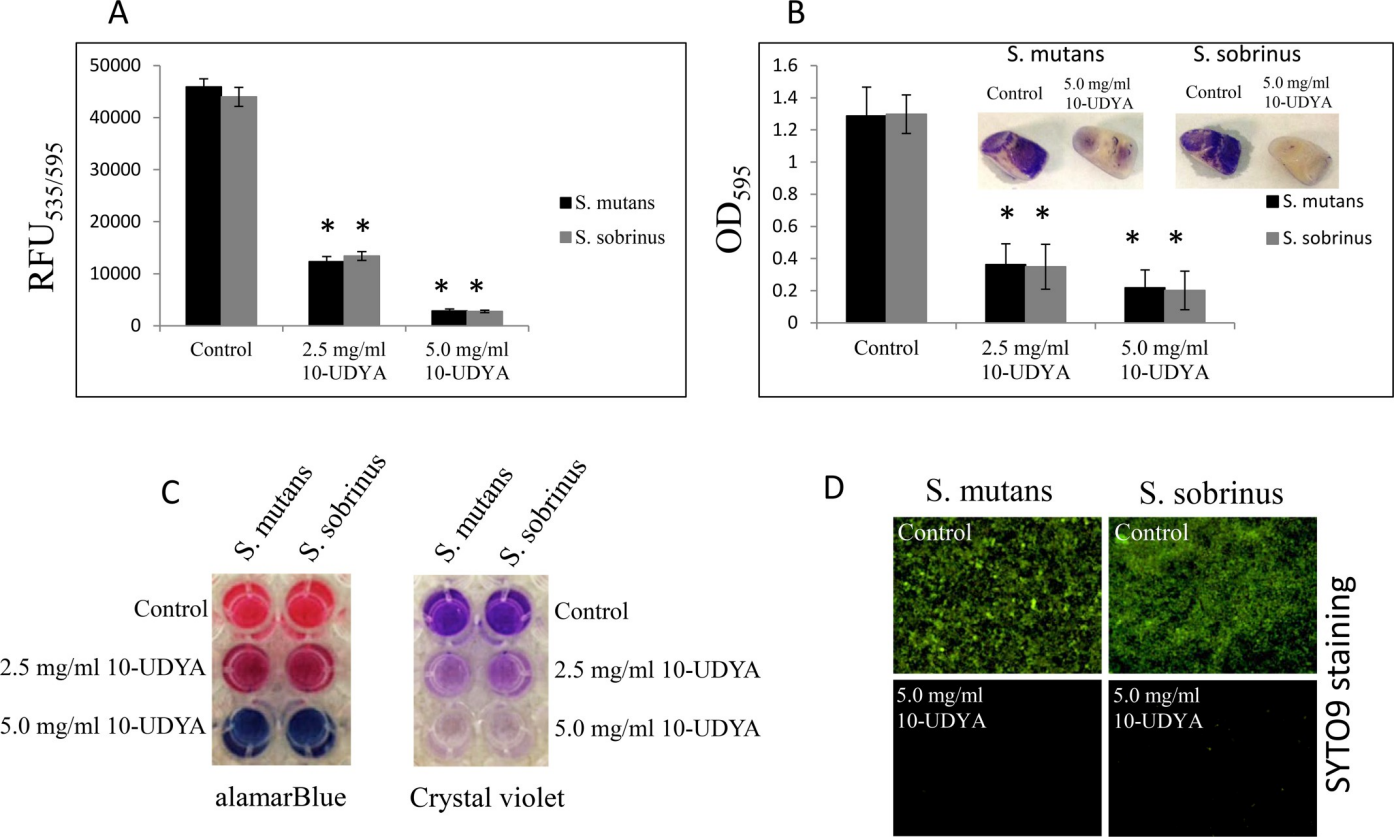
Effect of 10-undecynoic acid on the biofilm growth of *Streptococcus mutans* UA159 and *Streptococcus sobrinus* SL1. (A-C) MBIC values were determined on 96-well plates coated with human saliva after 24 h incubation with 10-undecynoic acid and assessed by alamarBlue and crystal violet. Inserts: representative images of biofilms of *Streptococcus* sp. formed on artificial teeth coated with human saliva after 24 h incubation period with 10-undecynoic acid. (D) Representative images of biofilms of *Streptococcus mutans* UA159 and *Streptococcus sobrinus* SL1 stained with SYTO9 after 24 h incubation period with 5.0 mg/ml of 10-undecynoic acid. Control– 0.1% DMSO, 10-UDYA– 10-undecynoic acid, * p<0.001 compared to control, RFU–relative fluorescence unit.

Testing 10-UDYA against the planktonic form of S*treptococcus* spp. showed growth inhibition at MIC 2.5 mg/ml and killing effect at MBC 5.0 mg/ml, after 24 h (Table 1). The kinetic evaluation revealed ~ 6-log_10_ CFU/ml decrease (99.9999% bactericidal effect of 10-UDYA against *S*. *mutans* UA159 and *S*. *sobrinus* SL1) after a 3 h incubation period, and ~ 2-log_10_ CFU/ml decrease (99% bactericidal effect of 10-UDYA against *S*. *mutans* UA159 and *S*. *sobrinus* SL1) after 1 h of incubation with 10-UDYA at 5.0 mg/ml. Observed bactericidal effect was found to be dose-dependent. As shown in Fig 1, there was no significant alternation in the growth pattern inhibition of *S*. *mutans* UA159 and *S*. *sobrinus* SL1. In addition to growth inhibition of *Streptococcus* spp. planktonic cultures, 10-UDYA inhibited the formation of their biofilms in BHI broth supplemented with 1% sucrose (MBIC_90_ 2.5 mg/ml) in 96-well plates coated with human saliva, whereas complete inhibition was achieved at 5.0 mg/ml (Table 1). Parallel experiments performed on artificial teeth revealed the same pattern. In the dose-dependent manner, 10-UDYA was also found to reduce the viability of culturing biofilms approximately 50–55% at 2.5 mg/ml. Reduced metabolic activity and, more profoundly, the formation of biofilms was observed when assessment was done by alamarBlue, SYTO9 staining, and crystal violet, with no differences in mean values noticed between tested *Streptococcus* spp. (Fig 2).

**Table 1:** Antibacterial effect of 10-UDYA tested against four oral *Streptococcus* spp.

Test parameters	10-undecynoic acid (mg/ml)					Amoxicillin (μg/ml)				
	MIC	MBC	MBIC_90_	MBBC	MBEC_50_	MIC	MBC	MBIC_90_	MBBC	MBEC_50_
*S*. *mutans* UA159	2.5	5.0	2.5	5.0	7.0	1.95[44]	2.0	7.8[44]	10	NE
*S*. *mutans* NCTC 10449	2.5	5.0	2.5	5.0	7.0	2.0	2.0	10	10	NE
*S*. *sobrinus* SL1	2.5	5.0	2.5	5.0	7.0	2.0	2.0	10	10	NE
*S*. *sobrinus* NIDR 6715–15	2.5	5.0	2.5	5.0	7.0	2.0	2.0	10	10	NE

Abbreviations: MIC—minimal inhibitory concentration, MBC—minimal bactericidal concentration, MBIC_90_—minimal biofilm inhibitory concentration that reduce the growth of biofilm at least in 90%, MBBC—minimal biofilm biocidal concentration, MBEC_50_—minimal biofilm eradication concentration that detach biofilm in at least 50%, NE—no effect/ susceptibility at the maximal tested concentration (i.e., 500 μg/ml).

**Fig 3:**
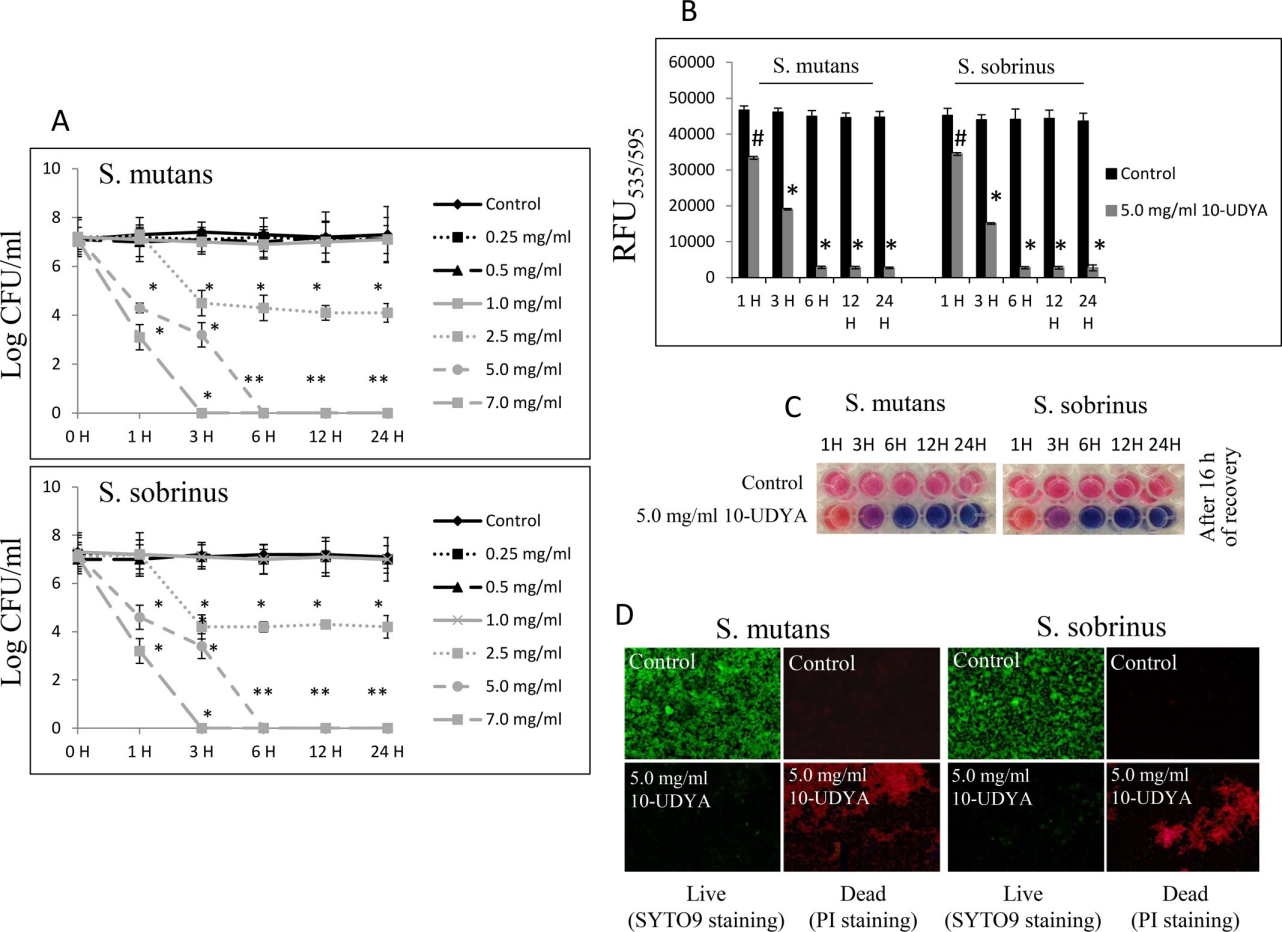
Biocidal effect of 10-undecynoic acid against the pre-existing biofilm of *Streptococcus mutans* UA159 and *Streptococcus sobrinus* SL1. (A-C) MBBC values were determined on 96-well plates coated with human saliva up to 24 h incubation period with 10-undecynoic acid and assessed by BHI agar plating method after 24 h and alamarBlue after 16 h of recovery period. (D) Representative images of *Streptococcus mutans* UA159 and *Streptococcus sobrinus* SL1 after 24 h incubation period with 5.0 mg/ml of 10-undecynoic acid stained with SYTO9/PI. Green fluorescence–live biofilm, red fluorescence–dead biofilm, control– 0.1% DMSO, 10-UDYA– 10-undecynoic acid, # p<0.05, * p<0.001 compared to control.

**Fig 4:**
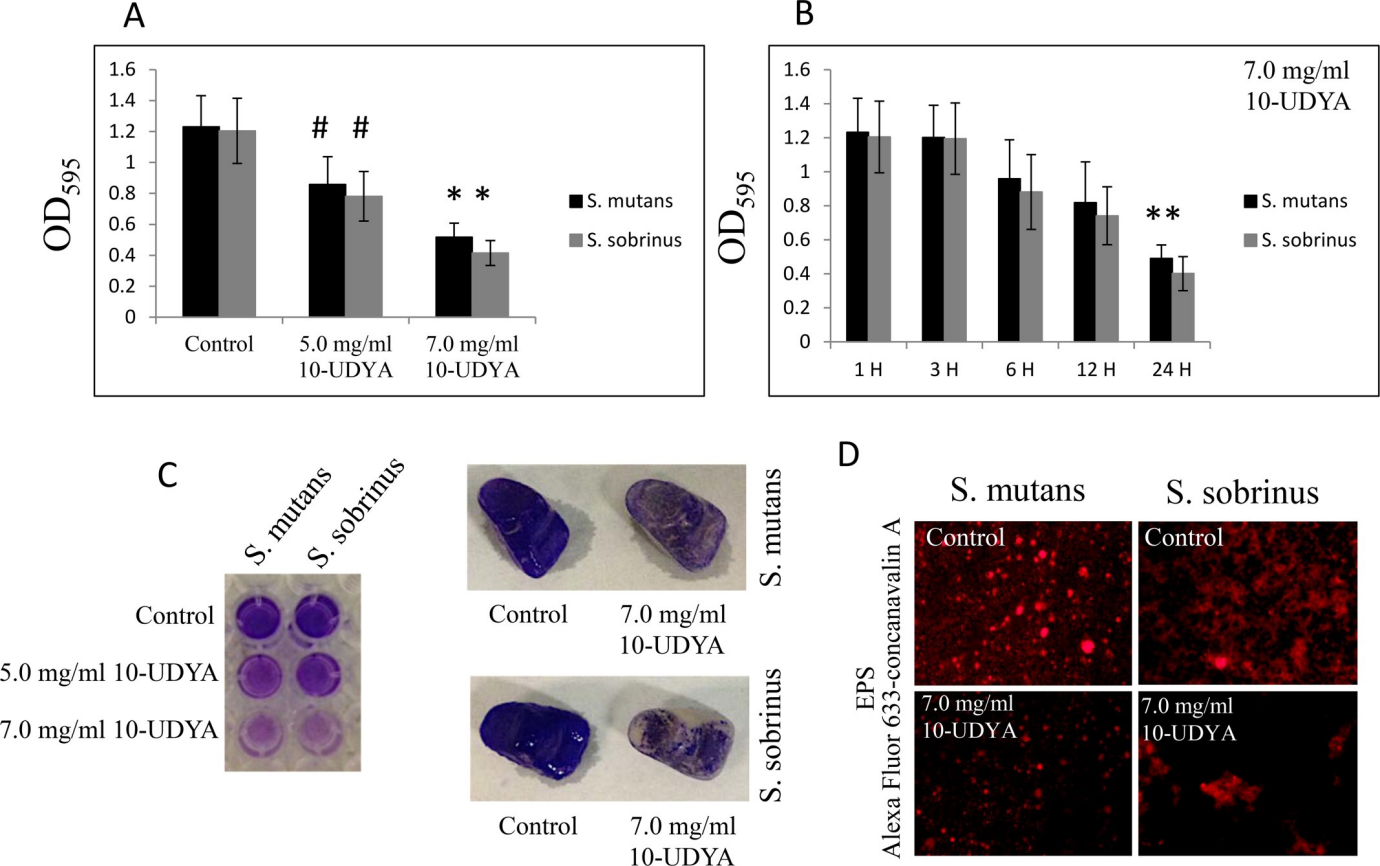
Effect of 10-undecynoic acid on the detachment of the pre-existing biofilm of *Streptococcus mutans* UA159 and *Streptococcus sobrinus* SL1. (A and C) MBEC values were determined by crystal violet staining on 96-well plates coated with human saliva after 24 h incubation with 10-undecynoic acid and artificial teeth coated with human saliva after 24 h. (B) Time-depended effect of 7.0 mg/ml 10-undecynoic acid assessed by crystal violet staining up to 24 h. (D) Representative images of EPS presence on pre-existing biofilm of *Streptococcus mutans* UA159 and *Streptococcus sobrinus* SL1 after 24 h incubation period with 7.0 mg/ml 10-undecynoic acid stained with Alexa Fluor 633-conjugated concanavalin A/red fluorescence. Control– 0.1% DMSO, 10-UDYA– 10-undecynoic acid, # p<0.05, * p<0.001 compared to control.

In the context of pre-existing biofilm growth in BHI broth supplemented with 1% sucrose in 96-well plates coated with human saliva, 10-UDYA caused dose- and time-dependent killing of pre-existing biofilms of *Streptococcus* spp. (MBBC 5.0 mg/ml) when assessed by standard BHI agar plating method (Fig 3). The experiment in which biofilms were first treated with 5.0 mg/ml 10-UDYA and then left for 16 h to allow the recovery, verified that this concentration killed at the rate of 99.9999% (~ 6-log_10_ CFU/ml decrease) after 6 h and with approximately 50% efficacy after 3 h (~ 2-log_10_ CFU/ml decrease), as measured by the alamarBlue method. Consequently, treatment with ≥ 5.0 mg/ml of 10-UDYA resulted in significantly reduced biomass of bacterial biofilms (Table 2). This effect seems to be dose-dependent since increased concentration of 10-UDYA to 7.0 mg/ml killed faster and augmented even more detachment of both *Streptococcus* spp. biofilms in a time-dependent manner compared to the control (p < 0.001); whereas, MIC concentration killing ability had a slower pace and the decrease in detachment reflected in biofilm biomass was moderate (Fig 4 and Table 2). The outcome of EPS staining and its quantitative analyses also revealed that, compared to the control, there was a significant reduction of EPS in 10-UDYA treated biofilms of *Streptococcus* spp. when EPS was detected by Alexa Fluor 633-conjugated concanavalin A, a carbohydrate-binding protein that selectively binds to a-mannopyranosyl and a-glucopyranosyl residues abundantly present in EPS matrix [45,46]. Pre-existing biofilms treated with 10-UDYA became thinner and looser in a dose-dependent fashion, that overall resulted in reduced biofilm biomass and less total protein as well as less water-soluble and water-insoluble polysaccharides. The observed downgrading trend began at MIC, but not at sub-MIC concentrations of 10-UDYA, and was similar for both tested *Streptococcus* spp. (Table 2).

**Table 2:** *Streptococcus* spp. biofilm composition after treatments with 10-UDYA.

Test parameters	*S*. *mutans* UA159				*S*. *sobrinus* SL1			
	Control	2.5 mg/ml	5.0 mg/ml	7.0 mg/ml	Control	2.5 mg/ml	5.0 mg/ml	7.0 mg/ml
Dry weight (mg/biofilm)	4.4±0.32	3.95±0.27	3.15±0.22*	2.15±0.12*	4.3±0.34	3.83±0.31	3.11±0.28*	2.04±0.22*
Total protein (mg/biofilm)	2.58±0.22	1.33±0.21*	1.25±0.24*	1.23±0.15*	2.53±0.25	1.33±0.20*	1.37±0.14*	1.28±0.17*
Soluble polysaccharides (mg/biofilm)	0.35±0.16	0.33±0.14	0.25±0.03*	0.23±0.05*	0.36±0.17	0.31±0.16	0.27±0.04*	0.22±0.07*
Insoluble polysaccharides (mg/biofilm)	1.14±0.12	0.96±0.21	0.65±0.22*	0.31±0.14*	1.17±0.25	0.91±0.20	0.57±0.15*	0.29±0.12*

* p<0.01–0.001 compared to control.

### Anti-adherent effect of 10-undecynoic acid against *Streptococcus mutans* UA159 *in vitro*

**Fig 5:**
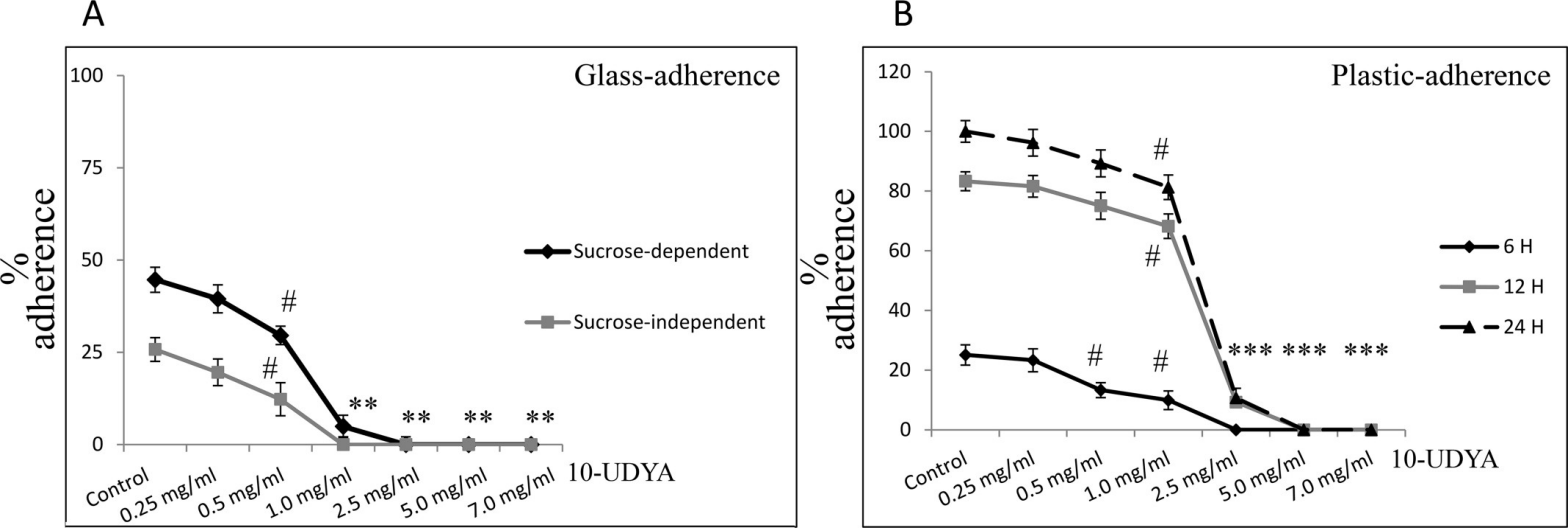
Effect of 10-undecynoic acid on the adherence of *Streptococcus mutans* UA159. (A) Glass-adherence in the absence (sucrose-independent) and presence of 1% sucrose (sucrose-dependent) was assessed by measuring turbidity at 600 nm after adding 0.5 M NaOH to reflect the number of adhered cells of *Streptococcus mutans* UA159. (B) Plastic-adherence in the presence of 1% sucrose (sucrose-dependent) was assessed at different time points by measuring absorbance of released CV dye at 595 nm from cells to reflect the number of adhered cells. Control– 0.1% DMSO, 10-UDYA– 10-undecynoic acid, # p<0.05, * p<0.001 compared to representative controls.

The inhibitory effect of different concentrations of 10-UDYA on adherence of *S*. *mutans* UA159 to glass and plastic surfaces is shown in Fig 5. 10-UDYA inhibited sucrose-independent and sucrose-dependent adherence of bacteria in a dose-dependent manner. The sub-MIC concentration of 10-UDYA (1 mg/ml) 100% inhibited sucrose-independent adherence of *S*. *mutans* UA159 to glass surface, whereas 0.5 mg/ml concentration of 10-UDYA inhibited the adherence of bacterial cell in ~ 50% (Fig 5A). Adherence to glass in the presence of 1% sucrose was evidently more apparent. Sucrose-dependent adherence of *S*. *mutans* UA159 was totally inhibited by MIC concentration (2.5 mg/ml) and approximately 80% by sub-MIC concentration (1 mg/ml). This effect was also tested at 6, 12, and 24 h on plastic plates to check whether it could adversely affect *S*. *mutans* UA159 biofilm formation at different phases of its biofilm growth: 6 h (adherent phase); 12 h (active accumulated phase); and 24 h (plateau accumulated phase) [47]. This study showed that the effect of 10-UDYA is concentration-dependent but not so much biofilm phase growth dependent (Fig 5B). The percentage of adherent bacteria gradually accumulated up to 24 h in the control and began gradually declining from the concentration of > 0.5 mg/ml 10-UDYA. Inhibition of biofilm formation by the 10-UDYA during the adherence phase, active accumulated phase, and the plateau accumulated phase was rather steady. At 6 h the adherence of bacterial cells at the MIC concentration of 10-UDYA was completely reduced, while at 12 h and 24 h the reduction was ~70–85% lower compared to the respective controls.

### Anti-acidogenic and anti-aciduric effect of 10-undecynoic acid against *Streptococcus mutans* UA159 *in vitro*

**Fig 6:**
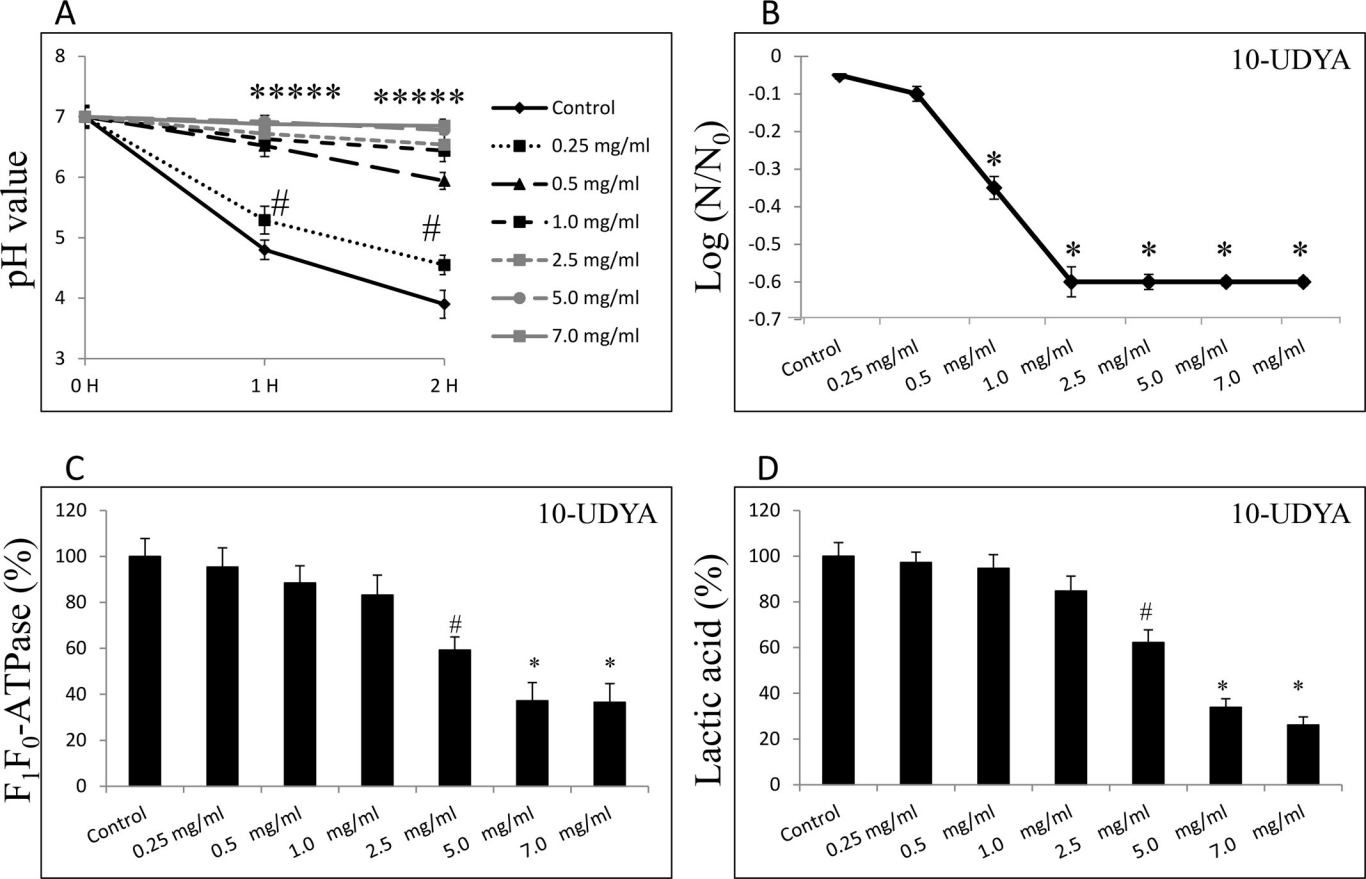
Effect of 10-undecynoic acid on acidogenicity and acidurity of *Streptococcus mutans* UA159. (A) Glycolytic acid production was determined by monitoring the pH decrease in sucrose solution (1% v/v) over a period of 2 h. (B) Acid tolerance was determined by measuring the survival rate of *Streptococcus mutans* UA159 at pH = 5.0 on BHI agar plates incubated for 24 h at 37°C. (C) Effects of 10-undecynoic acid on F_1_F_0_-ATPase of *Streptococcus mutans* UA159 was assessed as a relative enzymatic activity compared to untreated control. (D) Effects of 10-undecynoic acid on lactic acid production by *Streptococcus mutans* UA159 was assessed by determining its levels after 24 h as changes in absorbance measured at 570 nm. Control– 0.1% DMSO, 10-UDYA– 10-undecynoic acid, # p<0.05, * p<0.001 compared to control, N_0_ and N–CFU counts before (N_0,_ time = 0 h) and after 2 h (N_,_ time = 2 h) treatment in pH = 5.0 culture, respectively.

Acidogenicity was determined by monitoring the glycolytic pH drop and lactic acid production. Additionally, the experiments showing acidurity changes of bacterial cells upon 10-UDYA treatment were performed. As shown in Fig 6A, compared to control, glycolytic pH dropped slower and the terminal pH was higher in cultures of *S*. *mutans* UA159 treated with 10-UDYA in a dose-dependent manner, and were strongly significant at sub-MIC concentrations (≥ 0.5 mg/ml). Also, 10-UDYA reduced the acid tolerance of *S*. *mutans* UA159 to low pH beginning from sub-MIC levels (≥ 0.5 mg/ml). As shown in Fig 6B, the survival rate of *S*. *mutans* UA159 cells at pH = 5.0 began to be significantly lower compared to the control (p < 0.001) in a dose-dependent fashion. At the same time, 10-UDYA exhibited mild inhibitory effects on the activity of the F_1_F_0_-ATPase of *S*. *mutans* UA159 pre-existing biofilms that were reduced by ~ 30–35% at MIC concentration, and by ~ 55–60% at MBC concentration of 10-UDYA (Fig 6C). Assay determining lactic acid levels in pre-existing biofilms followed a similar trend as shown in Fig 6D.

## Discussion

Here for the first time we report about antibacterial properties of 10-undecynoic acid (10-UDYA) providing evidence about its bacteriostatic, bactericidal and anti-biofilm effects against MS. 10-UDYA is the terminal acetylenic analogue of lauric acid that specifically and irreversibly inactivates hepatic cytochrome P-450 enzymes *in vitro* but not *in vivo*, and which are responsible for *ω*-hydroxylation of lauric acid [48]. This inactivation, recognized as “suicidal process”, is NADPH- and time-dependent [29]. It has also been shown that administration of 10-undecynoic acid to rats causes a specific inhibition of *ω*-hydroxylation catalyzed by cytochrome P450 4A1, in particular, and that this is accompanied by a significant decrease in phosphatidylethanolamine content in the endoplasmic reticulum [49]. Cytochrome P-450 4A1 terminally oxidizes fatty acids resulting in formation of hydroxylated fatty acids, which are further converted to dicarboxylic acids. This so-called *ω*-oxidation, occurs in higher plants and animals, and also in bacteria and yeasts [49,50]. In this, acetylenic fatty acid analogs were found in nature, such as in seed oils of *Chrysanthemum corymbosum* and *Heisteria silvanii* [51,52]. There is a gap in knowledge with regard to scientific validation of the anti-bacterial, particularly, anti-biofilm and anti-virulence properties of such compounds, although there are some evidences of anti-microbial activities of fatty acids against gram-positive and gram-negative bacteria in general [53,54].

We found that conspicuous antibacterial effects follow the administration of 10-UDYA *in vitro*. We observed bacteriostatic and bactericidal potency of this compound against the planktonic form of cariogenic strains of *S*. *mutans* and *S*. *sobrinus* with the MIC of 2.5 mg/ml and MBC of 5.0 mg/ml after 3 h of its application. It was also of interest that 10-UDYA inhibited biofilm formation on the surface coated with human saliva with MBIC_90_ of 2.5 mg/ml with complete inhibition at 5.0 mg/ml. The biocidal concentration towards pre-existing biofilm was found to be the same (MBBC of 5.0 mg/ml after 6 h of treatment), whereas MBEC_50_ was established to be 7.0 mg/ml and this eradication effect of pre-existing biofilm showed to be dose- and time-dependent.

Observed inhibitory effect of 10-UDYA on biofilm development of *S*. *mutans* and *S*. *sobrinus* was dose- and time-dependent beginning at sub-MIC, greatly seen at MIC, with complete inhibition at MBC concentration. This decreased biofilm development in the presence of 10-UDYA was accompanied by altered bacterial growth or reduced metabolic activity. Moreover, strong inhibitory adherence effect of bacterial cells observed in the presence of high concentration of sucrose and sucrose-independent adherence was also reduced in a dose-dependent manner. According to Islam *et al*., the hydrophobic interaction of the bacteria with the glass surface is mostly responsible for sucrose-independent adherence; whereas, sucrose-dependent adherence is rather an effect of synthesis by bacteria gluey exopolysaccharides (glucan) from sucrose causing the clumping of the cells [55] and their synthesis and accumulation is in direct correlation with induction of dental caries by serotype c *S*. *mutans* [56]. The study of Crowley *et al*., showed that adhesin P1 of *S*. *mutans* is another important binding protein interacting directly with salivary agglutinin, which obstructs the aggregation and the none-sucrose adherence mechanisms *in vitro* [57]. Concentration-relevant reduction of sucrose-dependent and sucrose-independent adherence, even in the presence of sub-MIC and sub-MBC concentration of 10-UDAY, could be the result of two different “natures” of this fatty acid and explains observed alterations in the process of biofilm formation. Due to the lipophilic nature of 10-UDYA, it can be proposed that it crosses the plasma membrane, similar to the mechanism of action of other fatty acids [58,59]. Such inclusion in the membranes is projected to decrease hydrophobic lipid-lipid and lipid-protein interactions, and ultimately lower surface tensions [60,61]. Thus, this “surfactant-like” characteristic of this compound could be responsible for decreased hydrophobic interactions of bacteria with the glass surface resembling the mode of action of dispersants. Reducing the affinity of the organisms could also help in diminishing the voracity of the adherence of the biofilms to the plastic surface. On the other hand, since 10-UDYA inhibited polysaccharide-mediated adherence of the bacteria to the polystyrene plates in a concentration-dependent manner at the 6 h time point similar to 12 h and 24 h time points of incubation with the MIC concentrations of 10-UDYA, this would suggest its alternating potency in primary attachment of bacteria to the surface and lingering to the stage of active synthesis of glucan and plateau phase [62,63]. This compound also reduced also biomasses of bacterial biofilms in a concentration-dependent manner together with decreasing the amount of the total proteins (comprising GTFs and similar to amounts of water-soluble and water-insoluble polysaccharides) compared to control at MBC level. Since, the amount of total proteins but not the soluble and insoluble carbohydrates was found to be significantly lower at MIC level, we concluded that this compound, although effective, is not primarily targeting the production of polysaccharides. Additionally, we also found that the inhibition of water-soluble polysaccharides (via GTFC, which participate in formation of both soluble and insoluble glucans, and provide binding sites for oral bacteria) was to lesser extent inhibited by 10-UDYA than the water-insoluble polysaccharides (via GTFB, producing insoluble glucans and forming the scaffold of the EPS matrix) [13,64]. Fluorescence images depicting EPS biofilm undoubtedly showed disturbed altered structure with lack of integrity in the presence of MBC concentration of 10-UDYA. That would imply that there might yet be another factor that may be grounded in the lower viability of the bacterial cells, since decreased metabolic activity at MIC concentration of 10-UDYA was found to be approximately 50%. Nonetheless, this all depicts enhanced stress conditions for *S*. *mutans* upon 10-UDYA application which ultimately may result in altered development of biofilm as well as the architecture of already formed biofilm.

In addition, 10-UDYA revealed high biofilm penetrating potency and the excellent killing efficacy of pre-existing biofilm, another virulence factor of *S*. *mutans* that essentially attributes to its clinical complications [65]. Revealed restrained acidogenicity and aciduricity upon 10-UDYA treatment was also concurred. Glycolysis is the key pathway involved in acid production, in particular lactic acid. The results from the glycolytic pH drop showed a rather steady level of pH up to 2 h incubation with sub-MIC concentrations of 10-UDYA, suggesting the impairment in acidogenicity of *S*. *mutans*. It is also particularly noteworthy that pH sensitivity of *S*. *mutans* was distinctly altered causing a significantly reduced survival rate of *S*. *mutans* at pH = 5.0 even at sub-MIC concentrations of 10-UDYA. Moreover, MIC and MBC concentrations of 10-UDYA affected lactic acid levels and F_1_F_0_-ATPase activity that plays a crucial role in maintenance of sustainable pH for *S*. *mutans*. Since the link between the enzymatic activity of F_1_F_0_-ATPase and acid tolerance of plaque bacteria has been found [10,66–68], and considering that 10-UDYA might alter proper physiological organization of the bacterial membrane [58,69], it can be speculated that 10-UDYA may also affect the function of F_1_F_0_-ATPase, a membrane-bound protein, either directly or indirectly, and that could ultimately lead to restrained acidogenicity, reduced aciduricity, and finally killing effect. Interestingly, the interference of fatty acids with the electron transport system has been seen in yeasts and, as a result of their interactions with cell membranes, the disentanglement of the transmembrane proton gradients from the energy-requiring processes [70–73]. The changes in lactic acid level and F_1_F_0_-ATPase activity seen at MIC and MBC concentrations of 10-UDYA are consistent with detected decrease in metabolic activity. Thus, perhaps the involvement of other factors that have not been explored in this study, but are currently under investigation in our laboratory, could play a role in the killing process here.

In summary, our results showed that 10-UDYA acts as an effective antibacterial agent due to its inhibition of various cariogenic virulence factors of *S*. *mutans in vitro*, resulting in prime anti-adherence and biocidal properties, reflected in reduced acidogenicity, compromised aciduricity, and the effective ability to disrupt biofilms formation. 10-UDYA could be a promising compound for targeting biofilms of mutans streptococci, although more studies are needed to confirm this. According to U.S. Army Armament Research and Development Command, Chemical Systems Laboratory, NIOSH Exchange Chemicals. Vol. NX#07969, LD_50_ of 10-UNDY intravenously injected in mice is 32 mg/kg. More results from toxicological study are warranted to draw final a conclusion about the applicability of this compound, although in this case, incorporation of 10-UDYA to toothpastes or gels would rather be a “way of choice”. Also, the cost of its addition should be taken into account. That, however, needs to be determined on the specific and distinct bases.

Based on obtained results we conclude that 10-undecynoic acid might be a compound worth consideration in the development of future alternative or adjunctive antibacterial, especially, anti-biofilm preventive and/or therapeutic approaches.

## Ethical statement

Human saliva was used in this study that was purchased from Innovative Research (Novi, Michigan, USA).

## References

[1] Bagramian RA, Garcia-Godoy F, Volpe AR. The global increase in dental caries. A pending public health crisis. Am J Dent. 2009;22(1):3–8. 19281105

[2] Manji F, Dahlen G, Fejerskov O. Caries and Periodontitis: Contesting the Conventional Wisdom on Their Aetiology. Caries Res. 2018;52(6):548–64. 10.1159/000488948 29694978

[3] Sanz M, Beighton D, Curtis MA, Cury JA, Dige I, Dommisch H, et al Role of microbial biofilms in the maintenance of oral health and in the development of dental caries and periodontal diseases. Consensus report of group 1 of the Joint EFP/ORCA workshop on the boundaries between caries and periodontal disease. J Clin Periodontol. 2017;44(Suppl 18):S5–11. 28266109 10.1111/jcpe.12682

[4] Hamilton-Miller JM. Anti-cariogenic properties of tea (*Camellia sinensis*). J Med Microbiol. 2001;50(4):299–302. 10.1099/0022-1317-50-4-299 11289514

[5] Marcenes W, Kassebaum NJ, Bernabé E, Flaxman A, Naghavi M, Lopez A, et al Global burden of oral conditions in 1990–2010: a systematic analysis. J Dent Res. 2013;92(7):592–7. 10.1177/0022034513490168 23720570 PMC4484374

[6] Petersen PE. World Health Organization global policy for improvement of oral health—World Health Assembly 2007. Int Dent J. 2008;58(3):115–21. 18630105 10.1111/j.1875-595x.2008.tb00185.x

[7] Trahan L. Xylitol: a review of its action on mutans streptococci and dental plaque—its clinical significance. Int Dent J. 1995;45(1 Suppl 1):77–92. 7607748

[8] Forssten SD, Björklund M, Ouwehand AC. *Streptococcus mutans*, caries and simulation models. Nutrients. 2010;2(3):290–8. 10.3390/nu2030290 22254021 PMC3257652

[9] Hamada S, Slade HD. Biology, immunology, and cariogenicity of *Streptococcus mutans*. Microbiol Rev. 1980;44(2):331–84. 6446023 10.1128/mr.44.2.331-384.1980 PMC373181

[10] Belli WA, Marquis RE. Adaptation of *Streptococcus mutans* and *Enterococcus hirae* to acid stress in continuous culture. Appl Environ Microbiol. 199;57(4):1134–8. 1829347 10.1128/aem.57.4.1134-1138.1991 PMC182857

[11] Lemos JA, Quivey RG, Koo H, Abranches J. *Streptococcus mutans*: a new Gram-positive paradigm? Microbiology. 2013;159(Pt 3):436–45. 10.1099/mic.0.066134-0 23393147 PMC4083656

[12] Rudney JD. Saliva and dental plaque. Adv Dent Res. 2000;14:29–39. 10.1177/08959374000140010401 11842921

[13] Banas JA. Virulence properties of *Streptococcus mutans*. Front Biosci. 2004;9:1267–77. 14977543 10.2741/1305

[14] Bowen WH, Koo H. Biology of *Streptococcus mutans*-derived glucosyltransferases: role in extracellular matrix formation of cariogenic biofilms. Caries Res. 2011;45(1):69–86. 10.1159/000324598 21346355 PMC3068567

[15] Li Y, Burne RA. Regulation of the gtfBC and ftf genes of *Streptococcus mutans* in biofilms in response to pH and carbohydrate. Microbiology. 2001;147(Pt 10):2841–8. 10.1099/00221287-147-10-2841 11577162

[16] Yamashita Y, Bowen WH, Burne RA, Kuramitsu HK. Role of the *Streptococcus mutans* gtf genes in caries induction in the specific-pathogen-free rat model. Infect Immun. 1993;61(9):3811–7. 8359902 10.1128/iai.61.9.3811-3817.1993 PMC281081

[17] Krzyściak W, Jurczak A, Kościelniak D, Bystrowska B, Skalniak A. The virulence of *Streptococcus mutans* and the ability to form biofilms. Eur J Clin Microbiol Infect Dis. 2014;33(4):499–515. 10.1007/s10096-013-1993-7 24154653 PMC3953549

[18] Johnson CP, Gross SM, Hillman JD. Cariogenic potential *in vitro* in man and *in vivo* in the rat of lactate dehydrogenase mutants of *Streptococcus mutans*. Arch Oral Biol. 1980;25(11–12):707–13. 6943990 10.1016/0003-9969(80)90124-7

[19] Lemos JA, Burne RA. A model of efficiency: stress tolerance by *Streptococcus mutans*. Microbiology. 2008;154(Pt 11):3247–55. 10.1099/mic.0.2008/023770-0 18957579 PMC2627771

[20] Nascimento MM, Lemos JAC, Abranches J, Gonçalves RB, Burne RA. Adaptive acid tolerance response of *Streptococcus sobrinus*. J Bacteriol. 2004;186(19):6383–90. 10.1128/JB.186.19.6383-6390.2004 15375118 PMC516607

[21] Matsiu R, Cvitkovitch D. Acid tolerance mechanisms utilized by *Streptococcus mutans*. Future Microbiol. 2010;5(3):403–17. 10.2217/fmb.09.129 20210551 PMC2937171

[22] Chen A, Hillman JD, Duncan M. L-(+)-lactate dehydrogenase deficiency is lethal in *Streptococcus mutans*. J Bacteriol. 1994;176(5):1542–5. 8113201 10.1128/jb.176.5.1542-1545.1994 PMC205228

[23] Hillman JD, Chen A, Snoep JL. Genetic and physiological analysis of the lethal effect of L-(+)-lactate dehydrogenase deficiency in *Streptococcus mutans*: complementation by alcohol dehydrogenase from *Zymomonas mobilis*. Infect Immun. 1996;64(10):4319–23. 8926105 10.1128/iai.64.10.4319-4323.1996 PMC174373

[24] Hancock EB, Newell DH. Preventive strategies and supportive treatment. Periodontol 2000. 2001;25:59–76. 11155182 10.1034/j.1600-0757.2001.22250105.x

[25] Freires IA, Denny C, Benso B, de Alencar SM, Rosalen PL. Antibacterial activity of essential oils and their isolated constituents against cariogenic bacteria: A Systematic Review. Molecules. 2015;20(4):7329–58. 10.3390/molecules20047329 25911964 PMC6272492

[26] Gunsolley JC. Clinical efficacy of antimicrobial mouthrinses. J Dent. 2010;38(Suppl 1):S6–10. 20621242 10.1016/S0300-5712(10)70004-X

[27] Marsh PD. Sugar, fluoride, pH and microbial homeostasis in dental plaque. Proc Finn Dent Soc. 1991;87(4):515–25. 1775479

[28] Jeon J-G, Rosalen PL, Falsetta ML, Koo H. Natural products in caries research: current (limited) knowledge, challenges and future perspective. Caries Res. 2011;45(3):243–63. 10.1159/000327250 21576957 PMC3104868

[29] Ortiz de Montellano PR, Reich NO. Specific inactivation of hepatic fatty acid hydroxylases by acetylenic fatty acids. J Biol Chem. 1984;259(7):4136–41. 6706995

[30] Ccahuana-Vásquez RA, Cury JA. S. mutans biofilm model to evaluate antimicrobial substances and enamel demineralization. Braz Oral Res. 2010;24(2):135–41. 20658029 10.1590/s1806-83242010000200002

[31] Ajdić D, McShan WM, McLaughlin RE, Savić G, Chang J, Carson MB, et al Genome sequence of *Streptococcus mutans* UA159, a cariogenic dental pathogen. Proc Natl Acad Sci USA. 2002;99(22):14434–9. 10.1073/pnas.172501299 12397186 PMC137901

[32] Xu X, Zhou XD, Wu CD. The tea catechin epigallocatechin gallate suppresses cariogenic virulence factors of *Streptococcus mutans*. Antimicrob Agents Chemother. 2011;55(3):1229–36. 10.1128/AAC.01016-10 21149622 PMC3067078

[33] Pankey GA, Sabath LD. Clinical Relevance of Bacteriostatic versus Bactericidal Mechanisms of Action in the Treatment of Gram-Positive Bacterial Infections. Clin Infect Dis. 2004;38(6):864–70. 10.1086/381972 14999632

[34] Peedikayil FC. Antibiotics in Odontogenic Infections—An Update. J Antimicrob Agents. 2016;2(2):1–3.

[35] Lim JH, Song S-H, Park H-S, Lee JR, Lee S-M. Spontaneous detachment of *Streptococcus mutans* biofilm by synergistic effect between zwitterion and sugar alcohol. Sci Rep. 2017;7(1):8107 10.1038/s41598-017-08558-x 28808327 PMC5556044

[36] Decker E-M, Klein C, Schwindt D, von Ohle C. Metabolic activity of *Streptococcus mutans* biofilms and gene expression during exposure to xylitol and sucrose. Int J Oral Sci. 2014;6(4):195–204. 10.1038/ijos.2014.38 25059251 PMC5153587

[37] Koo H, Hayacibara MF, Schobel BD, Cury JA, Rosalen PL, Park YK, et al Inhibition of Str*eptococcus mutans* biofilm accumulation and polysaccharide production by apigenin and tt-farnesol. J Antimicrob Chemother. 2003;52(5):782–9. 10.1093/jac/dkg449 14563892

[38] He J, Hwang G, Liu Y, Gao L, Kilpatrick-Liverman L, Santarpia P, et al l-Arginine modifies the exopolysaccharide matrix and thwarts *Streptococcus mutans* outgrowth within mixed-Species oral biofilms. J Bacteriol. 2016;198(19):2651–61. 10.1128/JB.00021-16 27161116 PMC5019072

[39] Hamada S, Torii M, Kotani S, Tsuchitani Y. Adherence of *Streptococcus sanguis* clinical isolates to smooth surfaces and interactions of the isolates with *Streptococcus mutans* glucosyltransferase. Infect Immun. 1981;32(1):364–72. 6452415 10.1128/iai.32.1.364-372.1981 PMC350629

[40] Song J-H, Kim S-K, Chang K-W, Han S-K, Yi H-K, Jeon J-G. *In vitro* inhibitory effects of *Polygonum cuspidatum* on bacterial viability and virulence factors of *Streptococcus mutans* and *Streptococcus sobrinus*. Arch Oral Biol. 2006;51(12):1131–40. 10.1016/j.archoralbio.2006.06.011 16914113

[41] Svensäter G, Larsson UB, Greif EC, Cvitkovitch DG, Hamilton IR. Acid tolerance response and survival by oral bacteria. Oral Microbiol Immunol. 1997;12(5):266–73. 9467379 10.1111/j.1399-302x.1997.tb00390.x

[42] Belli WA, Buckley DH, Marquis RE. Weak acid effects and fluoride inhibition of glycolysis by *Streptococcus mutans* GS-5. Can J Microbiol. 1995;41(9):785–91. 7585355 10.1139/m95-108

[43] Muñoz MA, Balón M, Fernandez C. Direct determination of inorganic phosphorus in serum with a single reagent. Clin Chem. 1983;29(2):372–4. 6821948

[44] Clark SA, Vinson LA, Eckert G, Gregory RL. Effect of Commonly Prescribed Liquid Medications on *Streptococcus mutans* Biofilm. An *in vitro* study. J Clin Pediatr Dent. 2017;41(2):141–6. 10.17796/1053-4628-41.2.141 28288290

[45] Strathmann M, Wingender J, Flemming H-C. Application of fluorescently labelled lectins for the visualization and biochemical characterization of polysaccharides in biofilms of *Pseudomonas aeruginosa*. J Microbiol Methods. 2002;50(3):237–48. 12031574 10.1016/s0167-7012(02)00032-5

[46] Powell LC, Pritchard MF, Ferguson EL, Powell KA, Patel SU, Rye PD, et al Targeted disruption of the extracellular polymeric network of *Pseudomonas aeruginosa* biofilms by alginate oligosaccharides. NPJ Biofilms Microbiomes. 2018;4:13 10.1038/s41522-018-0056-3 29977590 PMC6026129

[47] Rukayadi Y, Hwang J-K. *In vitro* activity of xanthorrhizol against *Streptococcus mutans* biofilms. Lett Appl Microbiol. 2006;42(4):400–4. 10.1111/j.1472-765X.2006.01876.x 16599995

[48] CaJacob CA, Ortiz de Montellano PR. Mechanism-based in vivo inactivation of lauric acid hydroxylases. Biochemistry. 1986;25(16):4705–11. 3490272 10.1021/bi00364a038

[49] Lenart J, Pikuła S. 10-Undecynoic acid, an inhibitor of cytochrome P450 4A1, inhibits ethanolamine-specific phospholipid base exchange reaction in rat liver microsomes. Acta Biochim Pol. 1999;46(1):203–10. 10453996

[50] Van Bogaert INA, Groeneboer S, Saerens K, Soetaert W. The role of cytochrome P450 monooxygenases in microbial fatty acid metabolism. FEBS J. 2011;278(2):206–21. 10.1111/j.1742-4658.2010.07949.x 21156025

[51] Han L, Peng Y, Zhang Y, Chen W, Lin Y, Wang Q. Designing and Creating a Synthetic omega oxidation pathway in *Saccharomyces cerevisiae* enables production of medium-chain α, ω-dicarboxylic acids. Front Microbiol. 2017;8:2184 10.3389/fmicb.2017.02184 29163455 PMC5673993

[52] Tsevegsuren N, Christie WW, Lösel D. *Tanacetum (Chrysanthemum) corymbosum* seed oil—a rich source of a novel conjugated acetylenic acid. Lipids. 1998;33(7):723–7. 9688176 10.1007/s11745-998-0262-2

[53] Spitzer V, Tomberg W, Hartmann R, Aichholz R. Analysis of the seed oil of *Heisteria silvanii (Olacaceae)*-a rich source of a novel C18 acetylenic fatty acid. Lipids. 1997;32(11):1189–200. 9397405 10.1007/s11745-997-0153-6

[54] Fischer CL, Drake DR, Dawson DV, Blanchette DR, Brogden KA, Wertz PW. Antibacterial activity of sphingoid bases and fatty acids against Gram-positive and Gram-negative bacteria. Antimicrob Agents Chemother. 2012;56(3):1157–61. 10.1128/AAC.05151-11 22155833 PMC3294957

[55] Davies DG, Marques CNH. A fatty acid messenger is responsible for inducing dispersion in microbial biofilms. J Bacteriol. 2009;191(5):1393–403. 10.1128/JB.01214-08 19074399 PMC2648214

[56] Islam B, Khan SN, Haque I, Alam M, Mushfiq M, Khan AU. Novel anti-adherence activity of mulberry leaves: inhibition of *Streptococcus mutans* biofilm by 1-deoxynojirimycin isolated from Morus alba. J Antimicrob Chemother. 2008;62(4):751–7. 10.1093/jac/dkn253 18565974

[57] Crowley PJ, Brady LJ, Piacentini DA, Bleiweis AS. Identification of a salivary agglutinin-binding domain within cell surface adhesin P1 of *Streptococcus mutans*. Infect Immun. 1993; 61(4):1547–52. 8454362 10.1128/iai.61.4.1547-1552.1993 PMC281399

[58] Koga T, Asakawa H, Okahashi N, Hamada S. Sucrose-dependent cell adherence and cariogenicity of serotype c *Streptococcus mutans*. J Gen Microbiol. 1986;132(10):2873–83. 10.1099/00221287-132-10-2873 2957461

[59] Kabara JJ, Swieczkowski DM, Conley AJ, Truant JP. Fatty acids and derivatives as antimicrobial agents. Antimicrob Agents Chemother. 1972;2(1):23–8. 4670656 10.1128/aac.2.1.23 PMC444260

[60] Freese E, Sheu CW, Galliers E. Function of lipophilic acids as antimicrobial food additives. Nature. 1973;241(5388):321–5. 4633553 10.1038/241321a0

[61] Pang KY, Chang TL, Miller KW. On the coupling between anesthetic induced membrane fluidization and cation permeability in lipid vesicles. Mol Pharmacol. 1979;15(3):729–38. 91091

[62] Vanderkooi JM, Landesberg R, Selick H, McDonald GG. Interaction of general anesthetics with phospholipid vesicles and biological membranes. Biochim Biophys Acta. 1977;464(1):1–18. 831785 10.1016/0005-2736(77)90366-2

[63] Wen ZT, Burne RA. Functional genomics approach to identifying genes required for biofilm development by *Streptococcus mutans*. Appl Environ Microbiol. 2002;68(3):1196–203. 10.1128/AEM.68.3.1196-1203.2002 11872468 PMC123778

[64] Jefferson KK. What drives bacteria to produce a biofilm? FEMS Microbiol Lett. 2004;236(2):163–73. 15251193 10.1016/j.femsle.2004.06.005

[65] Xiao J, Klein MI, Falsetta ML, Lu B, Delahunty CM, Yates JR, et al The exopolysaccharide matrix modulates the interaction between 3D architecture and virulence of a mixed-species oral biofilm. PLoS Pathog. 2012;8(4):e1002623 10.1371/journal.ppat.1002623 22496649 PMC3320608

[66] Marsh PD. Are dental diseases examples of ecological catastrophes? Microbiology. 2003;149(Pt 2):279–94. 12624191 10.1099/mic.0.26082-0

[67] Hamilton IR, Buckley ND. Adaptation by *Streptococcus mutans* to acid tolerance. Oral Microbiol Immunol. 1991;6(2):65–71. 1658715 10.1111/j.1399-302x.1991.tb00453.x

[68] Bender GR, Marquis RE. Membrane ATPases and acid tolerance of *Actinomyces viscosus* and *Lactobacillus casei*. Appl Environ Microbiol. 1987;53(9):2124–8. 2445289 10.1128/aem.53.9.2124-2128.1987 PMC204068

[69] Bender GR, Sutton SV, Marquis RE. Acid tolerance, proton permeabilities, and membrane ATPases of oral streptococci. Infect Immun. 1986;53(2):331–8. 3015800 10.1128/iai.53.2.331-338.1986 PMC260879

[70] Hansch C, Glave WR. Structure-activity relationships in membrane-perturbing agents. Hemolytic, narcotic, and antibacterial compounds. Mol Pharmacol. 1971;7(3):337–54. 5106355

[71] Borst P, Loos JA, Christ EJ, Slater EC. Uncoupling activity of long-chain fatty acids. Biochim Biophys Acta. 1962;62:509–18. 13871487 10.1016/0006-3002(62)90232-9

[72] Corner TR. Synergism in the inhibition of *Bacillus subtilis* by combinations of lipophilic weak acids and fatty alcohols. Antimicrob Agents Chemother. 1981;19(6):1082–5. 6791586 10.1128/aac.19.6.1082 PMC181614

[73] Sheu CW, Salomon D, Simmons JL, Sreevalsan T, Freese E. Inhibitory effects of lipophilic acids and related compounds on bacteria and mammalian cells. Antimicrob Agents Chemother. 1975;7(3):349–63. 1137388 10.1128/aac.7.3.349 PMC429138

